# Phytochemicals-mediated production of hydrogen peroxide is crucial for high antibacterial activity of honeydew honey

**DOI:** 10.1038/s41598-018-27449-3

**Published:** 2018-06-13

**Authors:** Marcela Bucekova, Monika Buriova, Ladislav Pekarik, Viktor Majtan, Juraj Majtan

**Affiliations:** 1 Laboratory of Apidology and Apitherapy, Institute of Molecular Biology, Slovak Academy of Sciences, Dubravska cesta 21, 845 51 Bratislava, Slovakia; 2 Plant Science and Biodiversity Center, Slovak Academy of Sciences, Dubravska cesta 9, 845 23 Bratislava, Slovakia; 3 Department of Microbiology, Faculty of Medicine, Slovak Medical University, Limbova 12, 833 03 Bratislava, Slovakia

## Abstract

Honeydew honey is increasingly valued due to its pronounced antibacterial potential; however, the underlying mechanism and compounds responsible for the strong antibacterial activity of honeydew honey are still unknown. The aim of this study was to investigate the inhibition of bacterial growth of 23 honeydew honey samples. Activity of bee-derived glucose oxidase (GOX) enzyme, the content of defensin-1 (Def-1) and hydrogen peroxide (H_2_O_2_), and total polyphenol content were determined in the 23 honey samples. Our results demonstrated that antibacterial activity of honeydew honey was equivalent to medical-grade manuka and kanuka honey and was abolished by catalase. Although H_2_O_2_ is an important factor in the inhibition of bacterial growth, polyphenolic compounds and their interaction with H_2_O_2_ are the key factors responsible for high antibacterial activity of honeydew honey. In addition, our results indicated that the antibacterial activity of honeydew honey is not dependent on GOX-mediated production of H_2_O_2_ or the presence of Def-1.

## Introduction

Honeydew honey is produced by bees from sugar-rich secretions of trees and plants or exudates of plant-sucking insects (Hemiptera). In addition to its attractive sensorial characteristics, which differ from blossom honey, honeydew honey is valued due to its pronounced antibacterial potential^1^. The botanical origin of honeydew honey is from conifers or deciduous trees. A fir honeydew honey is one of the most known type of conifer honeydew honey in Europe^2^.

Honeydew honey possesses strong biological properties including antibacterial^1,3,4^, antibiofilm^5^, anti-inflammatory^6^, antioxidant^7^, as well as wound healing activity^8^. The antibacterial and antibiofilm activity of honeydew honey is comparable to manuka honey, which is currently used as medical-grade honey in clinical applications. Furthermore, a Slovak fir honeydew honey has been successfully clinically tested for the treatment of infected gluteo-femoral fistulas^9^, lower leg ulcers^10^, contact lens-induced corneal ulcers^11^, and as a prophylactic agent for endophthalmitis^12^.

The mechanism underlying the antibacterial and antibiofilm activity of honeydew honey has not been fully elucidated. It has been proposed that hydrogen peroxide (H_2_O_2_), produced by enzymatic conversion of glucose in diluted honey, and the bee-derived antibacterial peptide defensin-1 (Def-1) are considered the major components responsible for the antibacterial activity of natural honey, with the exception of manuka honey^13^. Although the levels of H_2_O_2_ in honey may differ between different types of honey, regardless of its botanical and geographical origin^14^, it has been suggested that honeydew honey produces higher amounts of H_2_O_2_ compared with nectar honey^15^. Honeydew honey is known for a high content of phenolic acids and flavonoids possessing antioxidant and pro-oxidant properties^16^. Thus, polyphenols, at concentration found in honeydew honey, could be involved in the generation of substantial amounts of H_2_O_2_^17,18^.

In the present study, we aimed to (i) determine the antibacterial activity of Slovak honeydew honey samples against the two most frequently isolated wound pathogens, (ii) investigate the mechanism of how honeydew honey inhibits bacterial growth, and (iii) elucidate the contribution of Def-1 and glucose oxidase (GOX)-mediated H_2_O_2_ antibacterial activity to the overall antibacterial activity of honeydew honey.

## Results

### Antibacterial activity of honeydew honey

**Figure 1:**
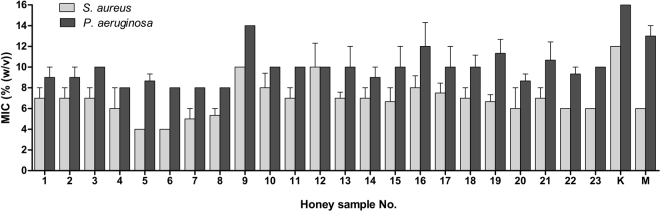
Antibacterial activity of honeydew honey samples (n = 23) and medical-grade manuka and kanuka honey against *Staphylococcus aureus* and *Pseudomonas aeruginosa* isolates. Activity was determined with a minimum inhibitory concentration (MIC) assay. The MIC was defined as the lowest concentration of honey solution (%) inhibiting bacterial growth. K, kanuka honey; M, manuka honey.

Minimum inhibitory concentration (MIC) values of 23 honeydew honey samples against the tested bacterial strains are shown in Fig. 1. Honey samples were more effective against of the Gram-positive *S. aureus* compared with the Gram-negative *P. aeruginosa*. For *S. aureus*, the MIC values of the honey samples ranged from 2.0 to 12.5%. The MICs of honey against *P. aeruginosa* ranged from 7.5 to 15%. Honey samples # 4–8 possessed the strongest antibacterial activity against the tested bacteria.

Besides MIC determination, a minimum bactericidal concentration (MBC) of honey samples was evaluated. The values of MIC and MBC were comparable and the overall inhibitory and bactericidal profiles of honey samples were identical (Fig. S1).

Kanuka and manuka honey, which are both used as medical-grade honeys, exhibited comparable or less effective antibacterial activity compared with the honeydew honey samples. Similar to honeydew honey, both types of New Zealand honey were more effective against *S. aureus* than *P. aeruginosa*.

### Comparison of GOX activity and H_2_O_2_ production in honey samples

**Figure 2:**
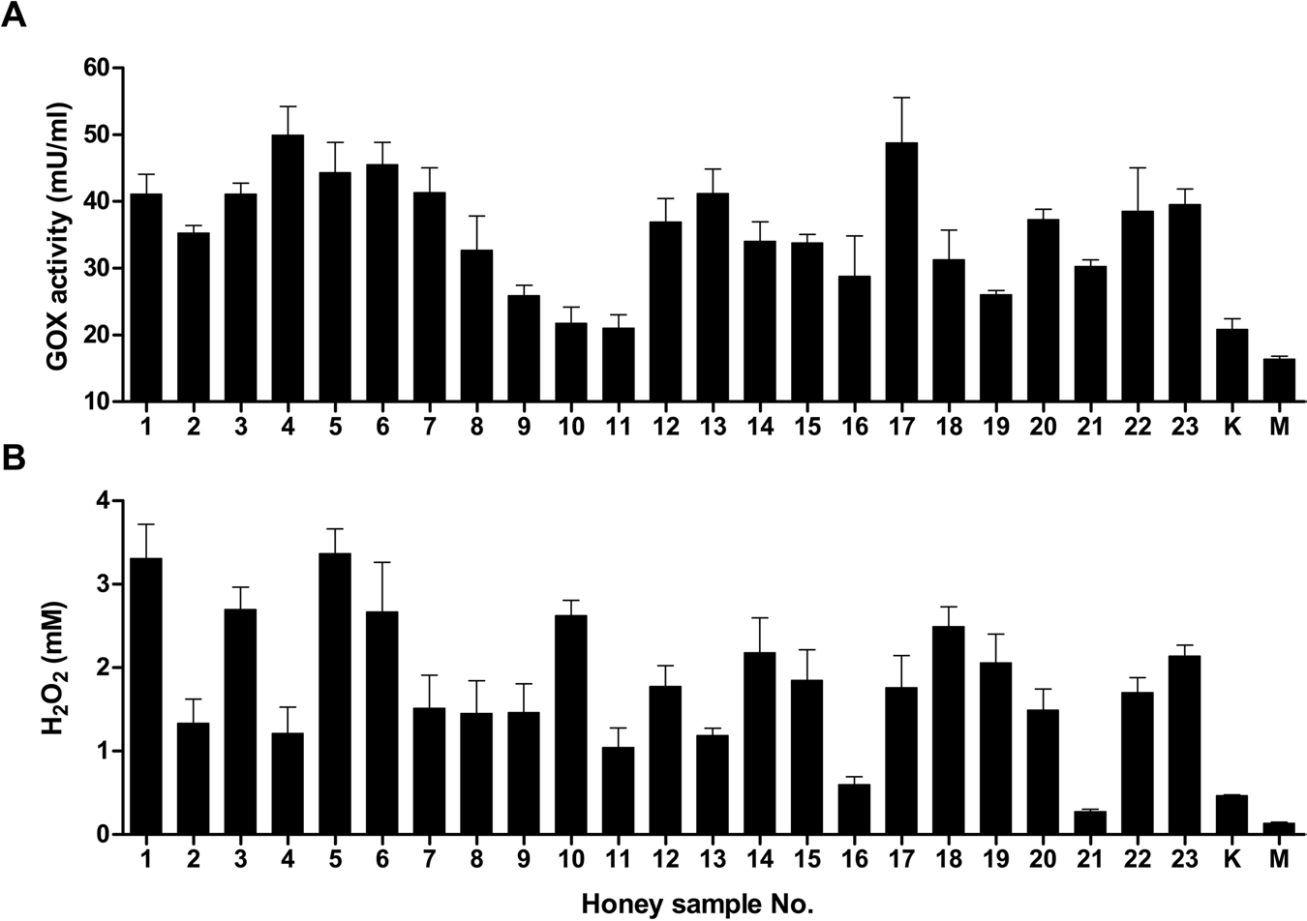
Glucose oxidase (GOX) activity and hydrogen peroxide (H_2_O_2_) production in honeydew honey samples (n = 23) and medical-grade manuka and kanuka honey. (**A**) GOX activity was determined in 20% (w/v) honey solutions with a GOX assay kit. (**B**) H_2_O_2_ production was measured in 40% (w/v) honey samples with a modified GOX assay kit. The data are expressed as the mean values with standard error of the mean (SEM).

The GOX activity determined in 20% honey solutions ranged from 21 to 50 mU/ml in all honeydew honey samples, with an average value of 35.7 mU/ml (Fig. 2A). The lowest enzymatic activity was monitored in both types of medical honey (<20 mU/ml), suggesting that methylglyoxal, present in both samples, may have structurally modified the GOX enzyme. The concentration of H_2_O_2_ in honeydew honey samples was determined in 40% honey solutions and ranged from 0.3 to 3.4 mM after 24 h incubation at 37 °C (Fig. 2B). The average value of H_2_O_2_ concentration was 1.8 mM. Two honeydew honey samples (#16 and #21) showed a weak production of H_2_O_2_, with values below 1 mM. As expected, kanuka and manuka honey accumulated the lowest levels of H_2_O_2_. No significant correlations were found between the values of GOX activity and H_2_O_2_ concentration among the honeydew honey samples (r = 0.26, *P* = 0.22). Therefore, we assumed that other compound(s) of botanical origin present in honeydew honey participate in the production of H_2_O_2_. Potential candidates responsible for the elevated levels of H_2_O_2_ include phenolic compounds.

### Total polyphenol and Def-1 content in honey samples

**Figure 3:**
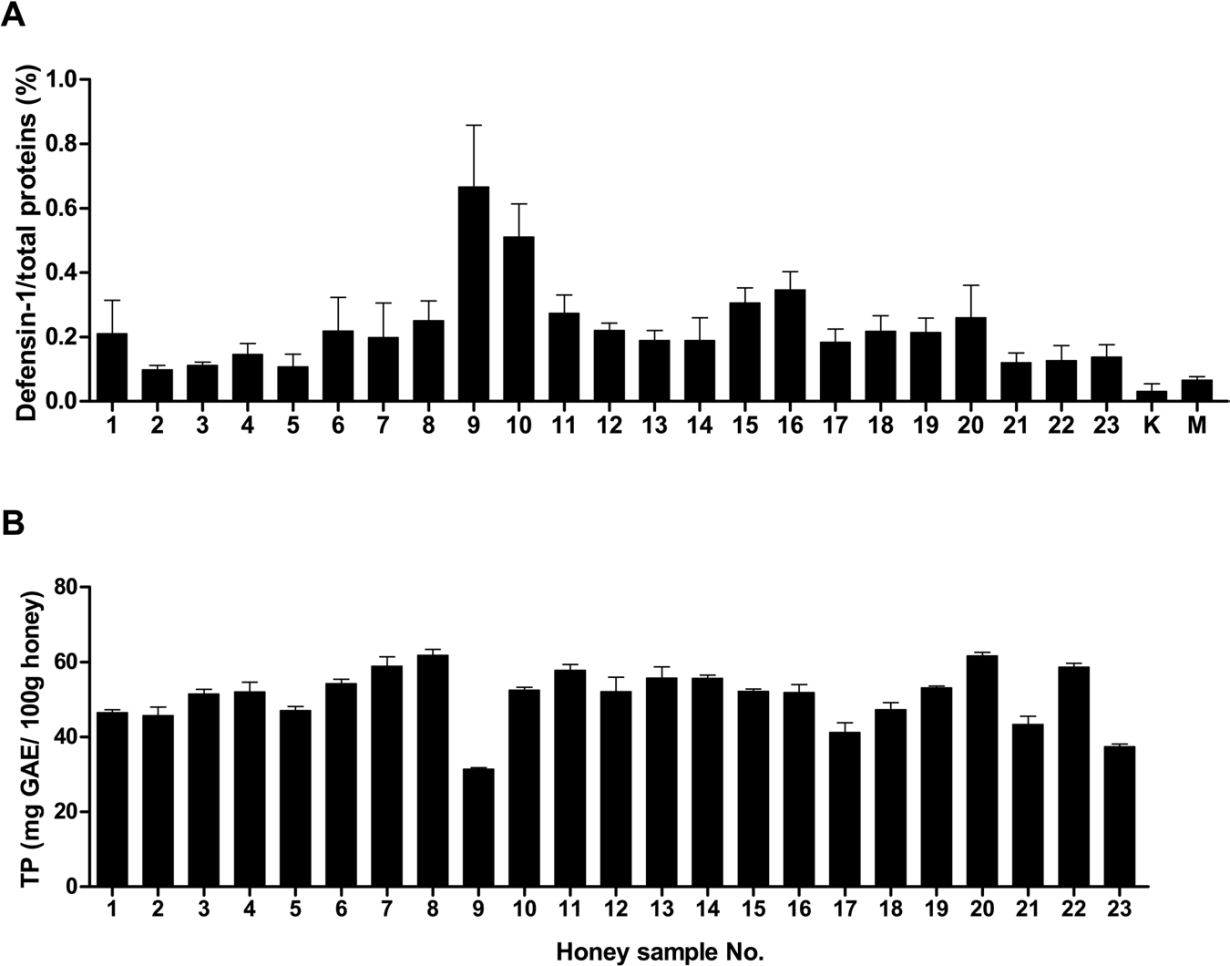
Content of defensin-1 (Def-1) and total polyphenol in honeydew honey samples (n = 23) and medical-grade manuka and kanuka honey. (**A**) Def-1 content was quantified in honey samples using indirect competitive enzyme-linked immunosorbent assay and the results were expressed as a percentage of total protein. (**B**) Total polyphenol (TP) content was determined with a Folin Ciocalteu Phenolic Content Quantification Assay Kit in 20% (w/v) honeydew honey solutions. Gallic acid (GAE) was used as the reference standard compound and results were expressed as GAE equivalents (mg/ml). Data are expressed as the mean values with standard error of the mean (SEM).

Def-1 is a key, but quantitatively variable, antibacterial factor in honey. Def-1 content was examined in the 2.5, 5 and 10% honey samples in PBS, using the newly developed competitive ELISA and the results were expressed as a percentage of total protein (Fig. 3A). The amount of Def-1 in each honey sample, including kanuka and manuka honey, is shown in Fig. 3A. The two honeydew honey samples #9 and #10 contained the highest amount of Def-1. Similar to the H_2_O_2_ production capacity, kanuka and Manuka honey had the lowest content of Def-1 among all the tested samples. Overall content of Def-1 in the honeydew honey samples did not correlate with their antibacterial activity against *S. aureus* (r = 0.17, *P* = 0.45). Def-1, at the concentration observed in the honeydew honey samples, did not exert its antibacterial activity at the MICs of the honeydew honey samples. The obtained data also suggest that the antibacterial action of kanuka and manuka honey dependeds on methylglyoxal or other unknown botanical compounds rather than bee-derived antibacterial factors.

A direct antibacterial activity of phenolic compounds has not been shown since their concentrations in honey are too low to determine overall activity. However, phenolic compounds may act in synergy via pro-oxidative action by generating elevated levels of H_2_O_2_ in honey, which mediates the inhibition of bacterial growth as a result of oxidative stress. Total phenolic content determined in the 20% (w/v) honeydew honey solutions is shown in Fig. 3B.

### Contribution of bee-derived antibacterial compounds to the antibacterial activity of honeydew honey

**Figure 4:**
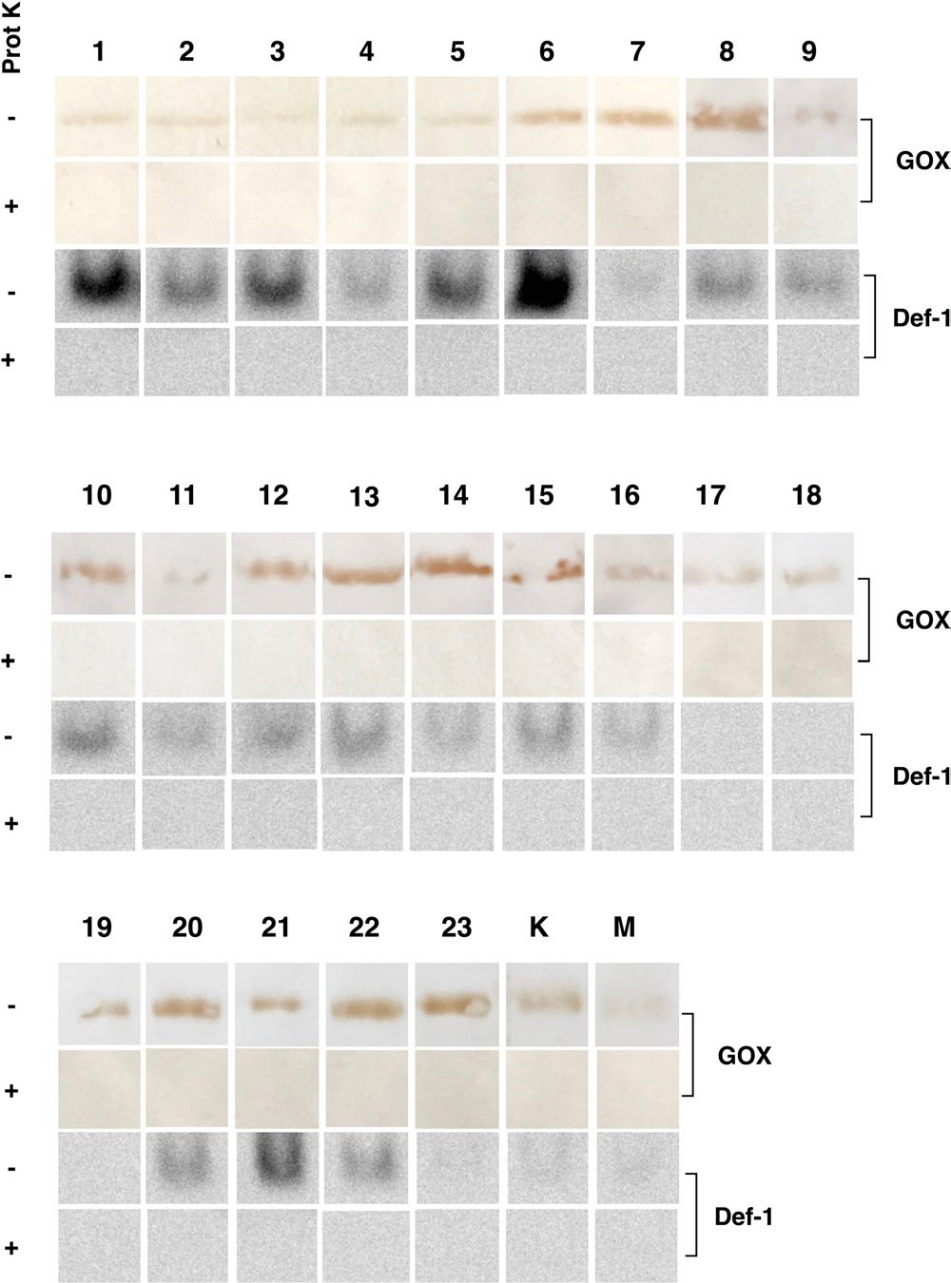
Detection of glucose oxidase (GOX) and defensin-1 (Def-1) in honeydew honey samples (n = 23) and medical-grade manuka and kanuka honey following proteinase K treatment by immunoblotting. Aliquots (15 μl) of 50% (w/v) honey solution with or without proteinase K were resolved by sodium dodecyl sulphate-polyacrylamide gel electrophoresis (SDS-PAGE) and 16.5% Tricine-SDS-PAGE. After semi-dry blotting procedure, the blocked membrane was incubated overnight with a rabbit polyclonal antibody against honeybee GOX or Def-1 (1:2000). Shown are cropped blots. (The blots with indicated cropping lines are shown in Supplementary Fig. S2). Immunoreactive bands were detected in solution containing dissolved SigmaFast 3,3-diaminobenzidine tablets (GOX) or detected using an enhanced chemiluminescence Immobilon Western kit (Def-1).

To investigate the contribution of bee-derived antibacterial compounds to the overall antibacterial activity of honeydew honey, samples were treated with a proteolytic enzyme, proteinase K. Upon proteinase K treatment, all proteinous content in the 50% (w/v) honey solutions was completely digested (data not shown). Untreated and proteinase K-treated honeydew samples were subjected to immunoblot analysis of GOX and Def-1. As expected, GOX and Def-1 were present in all untreated samples (Fig. 4). On the other hand, no immunoreactive bands for GOX and Def-1 were observed in treated samples, confirming the proteolytic effectiveness of proteinase K.

**Figure 5:**
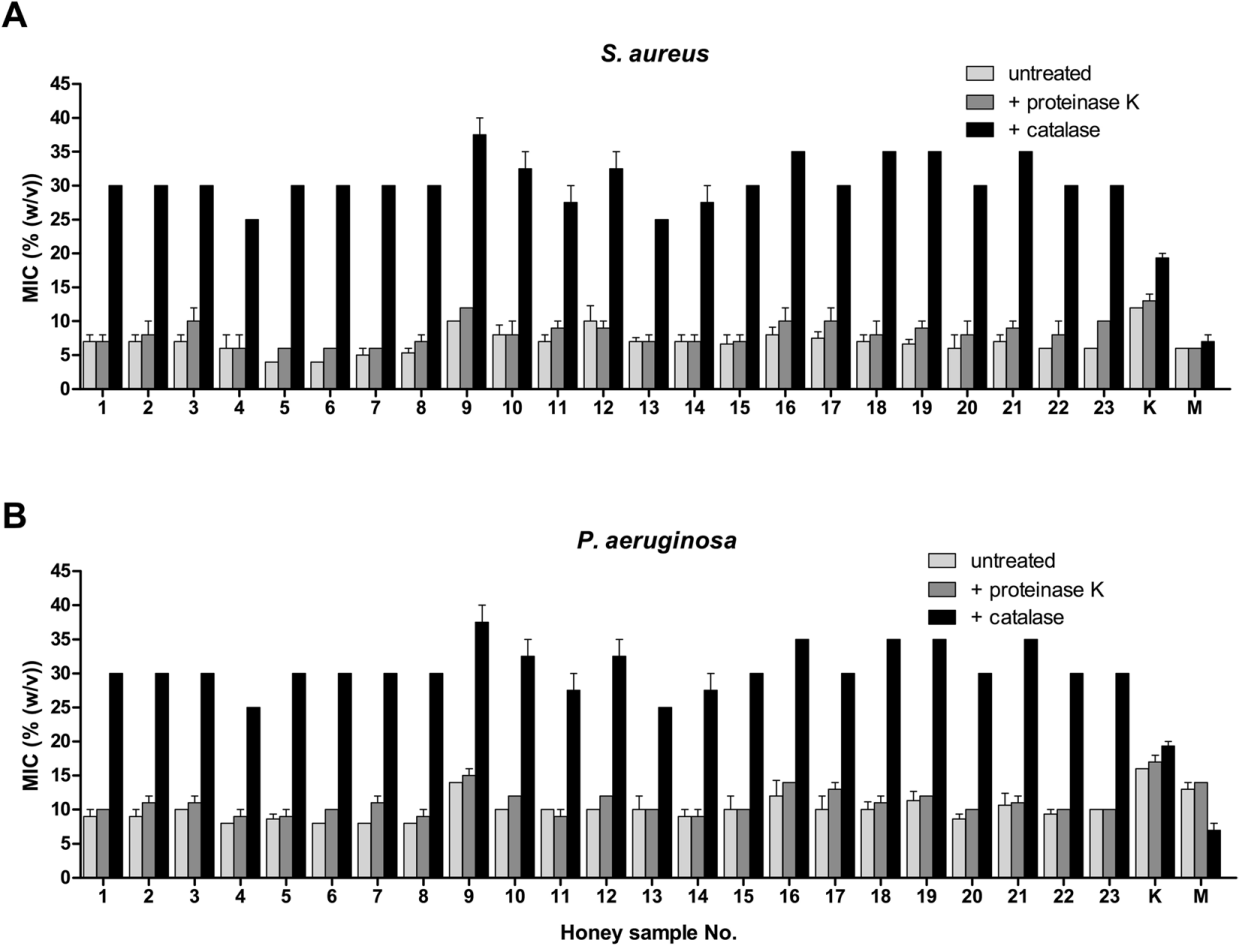
Antibacterial activity of honeydew honey samples (n = 23) and medical-grade manuka and kanuka honey following catalase and proteinase K treatment against (**A**) *Staphylococcus aureus* and (**B**) *Pseudomonas aeruginosa* isolates. The 50% (w/v) honey solutions were treated with catalase (2000–5000 U/mg protein) at a final concentration ranging from 1000 to 2500 U/ml at room temperature for 2 h or proteinase K (30 U/mg) at a final concentration of 50 μg/ml at 37 °C for 30 min. The antibacterial activity was determined with a minimum inhibitory concentration (MIC) assay. The MIC was defined as the lowest concentration of honey solution (%) inhibiting bacterial growth. K, kanuka honey; M, manuka honey.

MIC values were determined in untreated and proteinase K-treated honey samples. No significant changes were identified in the MIC values between untreated and proteinase-treated honey against both tested bacteria (Fig. 5), suggesting that bee-derived components, including GOX-mediated H_2_O_2_, are not responsible for the pronounced antibacterial activity of the honeydews honey. Although H_2_O_2_ is a major antibacterial compound of honeydew honey, its source might be solely of botanical origin.

### Role of H_2_O_2_ in honeydew honey antibacterial activity

**Figure 6:**
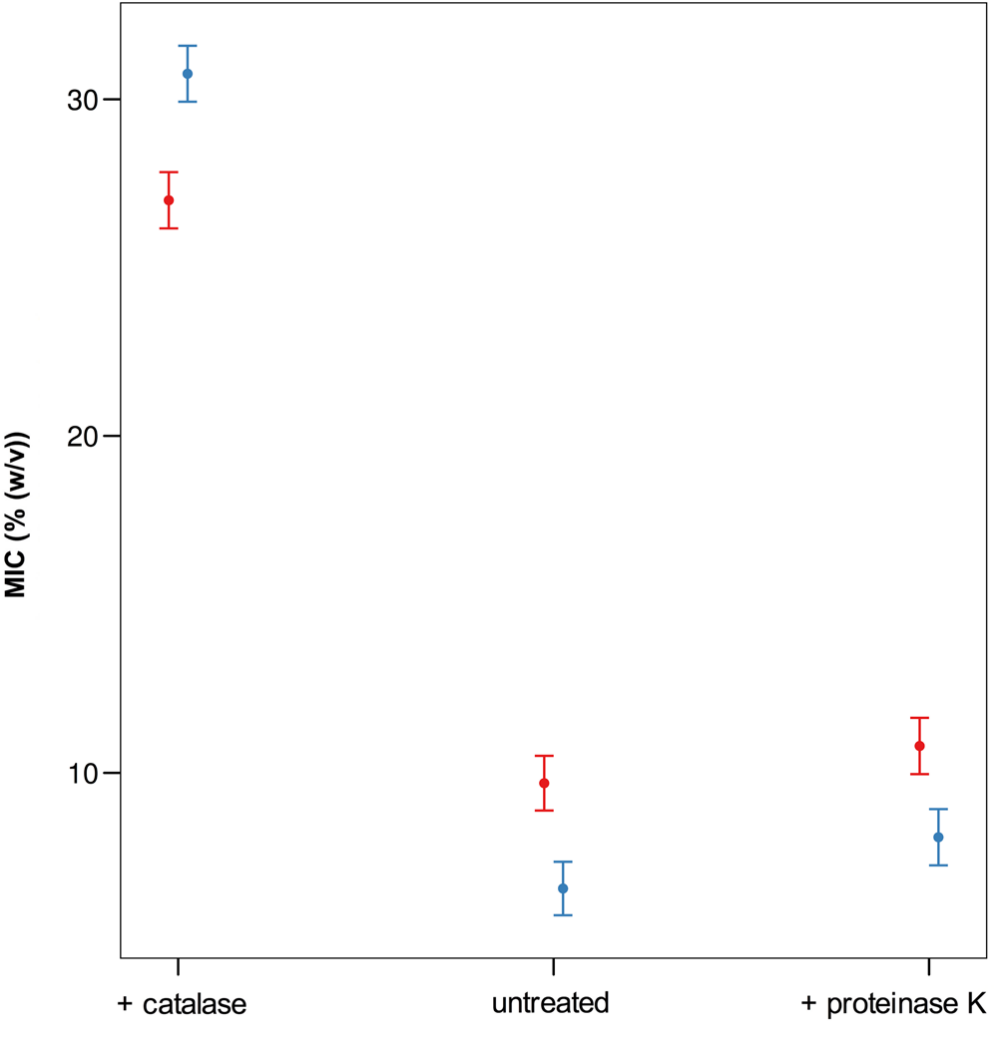
Graphical representation of the results of the general linear mixed model with response variable treatment type, bacterial species, and their two-way interaction. (*Pseudomonas aeruginosa – red and Staphylococcus aureus* – *blue)*.

To determine the role of H_2_O_2_ in honeydew honey antibacterial activity, 50% (w/v) honey solutions were treated with catalase for 2 h and MICs were determined. The MIC values of honey samples with or without catalase treatment against both tested bacteria are shown in Fig. 5. Catalase-treated samples had lower antibacterial activity with average MIC values of 30%. Furthermore, in seven samples, the MIC values were greater than 30% following catalase treatment. The effect of catalase on the MIC values in all honeydew honey samples was highly significant (NumDF = 2, DenDF = 268.43, F = 2136.38, *P* < 0.001) and predicted values were three times larger (Fig. 6) compared with untreated and protease K-treated samples. Interestingly, statistically significant changes (NumDF = 1, DenDF = 268.27, F = 8.52, *P* = 0.004)) were observed following catalase treatment between *S. aureus* and *P. aeruginosa* (Fig. 6). *S. aureus* was more resistant to the honey solution after the removal of H_2_O_2_ compared with *P. aeruginosa*.

Interestingly, the MIC of kanuka honey following catalase treatment was also changed, suggesting that the antibacterial activity of kanuka honey is, at least in part, due to the generation of H_2_O_2_. As expected, no remarkable changes in antibacterial activity were observed in manuka honey. Surprisingly, the antibacterial activity of manuka honey against *P. aeruginosa* following catalase treatment was even higher than without enzyme treatment.

Despite the fact that the antibacterial activities of honeydew honey samples were comparable, H_2_O_2_ production was not uniform. Statistical analysis did not show any correlation between antibacterial activity and the level of H_2_O_2_ in the different honey samples (r = −0.38, *P* = 0.08 and r = −0.03, *P* = 0.89). The honey samples with the highest level of H_2_O_2_ were not the most active.

## Discussion

Honey is increasingly valued for its antibacterial and wound healing activity and effectiveness as a treatment of chronic wounds and burns. However, every natural honey exhibits a certain antibacterial activity and only those with a strong and relatively stable antibacterial activity have been selected for medical and clinical use. In this study, we focused on honeydew honey as a source of potential medical-grade honey to elucidate its antibacterial activity and to investigate the role of bee-derived antibacterial components on its overall antibacterial activity.

A plethora of studies has demonstrated the important role of Def-1 and H_2_O_2_ in the antibacterial activity of honey.

Def-1 is an antibacterial peptide belonging to the insect defensin group that is composed of 51 amino acids with a molecular weight of 5.52 kDa. Bee Def-1 is effective against Gram-positive bacteria^19–21^; however, some studies using recombinant Def-1 have also reported its activity against Gram-negative bacteria including *P. aeruginosa* and *Salmonella choleraesuis*^22,23^. According to our recent study^24^, Def-1 is a common but quantitatively variable component in honey and its levels in different blossom honey samples are correlated with antibacterial activity against *S. aureus*. Recently, Def-1 has also been shown to be an immunomodualtor in wound healing where it positively contributes to cutaneous wound closure^25^. In the present study, the amount of Def-1 in honeydew honey did not correlate with the overall antibacterial activity of samples against *S. aureus*, suggesting that its contribution at low dilutions (5–10%) is negligible.

The most important antibacterial factor in honey is H_2_O_2_, which is produced by GOX-mediated conversion of glucose to gluconic acid under aerobic conditions in diluted honey^26^. Therefore, GOX plays an important role in the generation of H_2_O_2_^15^. Although H_2_O_2_ at concentrations found in honey does not kill bacteria, it is able to interact with bacterial cell proliferative signals, and thus affects bacterial growth even at high dilutions of honey^27^. Some researchers have suggested that the levels of H_2_O_2_ in honey may differ between types of honey regardless of botanical and geographical origin^14^. A few studies have attempted to determine the concentration of H_2_O_2_ in different honey samples and to evaluate its effect on the overall antibacterial activity^15,27–30^. Many studies have shown a greater correlation between antibacterial activity against *S. aureus* and the amount of H_2_O_2_ in honey samples. In the present study, there was no obvious relationship between the level of H_2_O_2_ and the antibacterial activity of the honeydew honey samples; the honey samples with the highest level of H_2_O_2_ were not the most active. Antibacterial activity was comparable among all honeydew honey samples despite variations in the concentration of H_2_O_2_. In addition, the removal of H_2_O_2_ by catalase did alter the antibacterial activity, as the activity following catalase treatment was comparable among all the honeydew honey samples. Similarly, Roshan and co-workers (2017) found no relationship between the concentration of H_2_O_2_ and overall antibacterial activity of eight different Australian honey samples. Researchers have suggested that low levels of H_2_O_2_ in different types of honey may be a result of the presence of phytochemicals other than methylglyoxal that might contribute to overall activity^30^.

We assume that plant-derived polyphenolic compounds, often reported in honey samples, may contribute to or modulate antibacterial activity. In the presence of transition metal ions (Cu and Fe) and peroxides, polyphenols, well-known dietary antioxidants, can act as pro-oxidants by accelerating hydroxyl radical formation and oxidative strand breakage in DNA^31^. In fact, polyphenols work in two ways to promote antibacterial activity: by directly producing H_2_O_2_, and by reducing Fe (III) to Fe (II), which triggers the Fenton reaction to create more potent reactive oxygen species such as hydroxyl radicals. A key factor in determining whether polyphenolic compounds exhibit antioxidative or antibacterial properties is pH value^32^. In weakly alkaline conditions (pH 7.0–8.0), polyphenols can exhibit pro-oxidative activity and inhibit bacterial growth. In the present study, the determination of honey MIC values and H_2_O_2_ concentration was carried out at pH 7.3 and 7.0, respectively. In these experimental conditions, it is obvious that the polyphenols in honeydew honey may act in synergy and inhibit bacterial growth. As mentioned, we measured the accumulation of H_2_O_2_ in diluted honey samples, but found no correlation between the concentration of H_2_O_2_ and antibacterial activity. It is likely that besides accumulation of H_2_O_2_, polyphenol-mediated production of hydroxyl radicals in honeydew honey significantly affects the overall antibacterial activity. Moreover, it has been shown that the chemical interaction of honey polyphenols with H_2_O_2_ results in the generation of products responsible for the degradation of bacterial DNA^33^. Interestingly, H_2_O_2_ alone is not able to induce DNA breaks but is somehow involved in this process. Although H_2_O_2_ is necessary for accelerating the auto-oxidation of polyphenols via hydrolysis and serves as a source of hydroxyl radicals, polyphenol concentration is a critical factor for antibacterial activity.

Total polyphenol content was reported to be high in other studies of honeydew honey samples^7,34,35^. In the present study, the total polyphenol content did not differ substantially between the tested honeydew honey samples (with the exception of sample #15), with an average value of 50 mg GAE/100 g of honey. It will be important to determine the critical concentration limit of total polyphenols for their behavior as pro-oxidant agents.

Several studies have attempted to identify biologically-active polyphenols and flavonoids from honeydew honey of different botanical and geographical origin^6,36,37^. Ferulic acid, quercetin, kaempherol and particular p-coumaric acid were the most abundant polyphenols in honeydew honeys. In a very recent studies^38^, combination of p-coumaric acid with bacteriocin showed synergistic effects against planktonic cells of food-borne bacteria. Similarly, combination of plant polyphenols and H_2_O_2_ was shown to be more effective against bacteria than H_2_O_2_ alone and can induce oxidative stress-related responses in bacteria^32^. It has been hypothesized that antibacterial effect of plant polyphenols is linked to polyphenols-induced H_2_O_2_ and an increase of endogenous reactive oxygen species generation^39^.

Until now, no study had been conducted to determine the role of bee-derived antibacterial compounds in honeydew honey. Here, we showed that the antibacterial activity of honeydew honey is not dependent on GOX-mediated production of H_2_O_2_ or the presence of Def-1. This is an interesting observation in the light of current knowledge regarding the antibacterial properties of honey. The antibacterial potential of honeydew honey is therefore not associated with the production capacity of GOX and Def-1 in particular bee colonies, which has a great impact on the biological properties of honey.

In conclusion, the tested honeydew honey samples showed equivalent or, in some cases, higher antibacterial activity compared with medical-grade kanuka and manuka honey. This strong antibacterial activity was abolished by treatment with catalase. Although H_2_O_2_ is an important factor in the inhibition of bacterial growth, phytochemicals such as polyphenolic compounds and their interaction with H_2_O_2_ are the key factors responsible for the antibacterial activity of honeydew honey. In addition, our results indicate that the antibacterial activity of honeydew honey is not dependent on GOX-mediated production of H_2_O_2_ or the presence of Def-1.

## Materials and Methods

### Honey samples

Honey samples (n = 28) collected in 2016 were received from beekeepers from several regions of Slovakia. The samples were selected according to their electrical conductivity value (Codex Alimentarius, Bogdanov 2002). Five samples were excluded from the study, as they did not meet the criteria for honeydew honey. Overall, 23 honeydew honey samples were used for further analysis. The honey samples were also compared to medical grade honey, Manuka Honey MGO 550 purchased from Manuka Health (New Zealand), and raw kanuka honey (HLKN1, No. 5054), kindly provided by Dr. Shaun Holt (honeylab, New Zealand).

### Microorganisms

The antibacterial activity of honey samples was assessed against the isolates *Pseudomonas aeruginosa* CCM1960 and *Staphylococcus aureus* CCM4223, obtained from the Department of Medical Microbiology, Slovak Medical University (Bratislava, Slovakia). These bacterial isolates are the two most frequently isolated pathogens from wounds.

### Determination of honey antibacterial activity

The antibacterial efficacy of the honey samples was evaluated with a minimum inhibitory concentration (MIC) assay as described by Bucekova *et al*. (2014). Briefly, overnight bacterial culture was suspended in phosphate-buffered saline (PBS), pH 7.2, and the turbidity of the suspension was adjusted to 10^8^ colony-forming unit (CFU)/ml and diluted with Mueller-Hinton broth (MHB) medium (pH 7.3 ± 0.1) to a final concentration of 10^6^ CFU/ml. Then, 10- μl aliquots of suspension were inoculated into each well of sterile 96-well polystyrene plates (Sarstedt, Germany). The final volume in each well was 100 μl, consisting of 90 μl of sterile medium or diluted honey and 10 μl of bacterial suspension. After 18 h of incubation at 37 °C, bacterial growth inhibition was determined by monitoring the optical density at 490 nm. The MIC was defined as the lowest concentration of honey inhibiting bacterial growth. All tests were performed in triplicate and repeated three times.

Serial dilutions of each honey sample were prepared from a 50% (w/v) honey solution, resulting in final concentrations of 45, 35, 30, 25, 20, 18, 16, 14, 12, 10, 8, 6, and 4%.

Besides MIC determination, MBC was evaluated in honey samples^40^. The viability of bacteria in wells with no turbidity was determined by spreading 100 µl onto an MHB agar plate and incubating at 37 °C for 24 h. The lowest honey that resulted in no survival of viable bacteria was recorded as MBC.

### Enzymatic treatment of honey samples with catalase and proteinase K

The 50% (w/v) honey solutions were treated with catalase (2000–5000 U/mg protein; Sigma-Aldrich, UK) at a final concentration ranging from 1000–2500 U/ml at room temperature for 2 h or proteinase K (30 U/mg; Promega, WI, USA) at a final concentration of 50 μg/ml at 37 °C for 30 min. Catalase- and proteinase K-treated honey samples were then used in the antibacterial assay to determine MIC values against *S. aureus* and *P. aeruginosa*.

To determine the effectiveness of both enzymes, the accumulation of H_2_O_2_ was measured in catalase-treated samples after 24 h incubation at 37 °C and the presence of GOX and Def-1 in proteinase K-treated samples were determined by immunoblot analysis (see 2.9.).

### Determination of GOX activity

The bee-derived GOX activity was determined by with a Megazyme GOX assay kit (Megazyme International Ireland Ltd., Bray, Ireland), which is based on the oxidative catalysis of β-D-glucose to D-glucono-δ-lactone, with the concurrent release of H_2_O_2_. The resultant H_2_O_2_ reacts with p-hydroxybenzoic acid and 4-aminoantipyrine in the presence of peroxidase to form a quinoneimine dye complex, which has a strong absorbance at 510 nm. The enzyme activity was determined in 20% (w/v) honey solutions in a 96-well microplate according to the manufacturer’s instructions.

### Determination of H_2_O_2_ concentration

The H_2_O_2_ concentration in the honey samples was determined with a Megazyme GOX assay kit (Megazyme International Ireland Ltd), which is based on H_2_O_2_ release. For a standard, H_2_O_2_ diluted to 9.8–312.5 μM was used. Briefly, 40% (w/v) honey solutions were incubated at 37 °C for 24 h. Each honey sample and standard was tested in duplicate in a 96-well microplate and the absorbance was measured at 510 nm using Synergy HT microplate reader (BioTek Instruments, VT, USA).

### Quantification of Def-1

Bee-derived peptide Def-1 was quantified as described by Valachova *et al*. (2016). Briefly, serial dilutions of each honey sample were prepared from a 50% (w/v) solution of honey, resulting in final concentrations of 2.5, 5, and 10%. An enzyme-linked immunosorbent assay (ELISA) was performed using a rabbit polyclonal anti-honeybee Def-1 antibody raised against a synthetic peptide corresponding to the C-terminus of bee Def-1 (CRKTSFKDLWDKRFG)^24^. The amount of Def-1 measured was expressed as a percentage of total proteins, which were measured using the Quick Start Bradford Protein Assay (Bio-Rad, CA, USA) according to the manufacturer’s instructions. All measurements were performed in triplicate and repeated three times.

### Total phenolic content

Total phenolic content was determined with a Folin Ciocalteu Phenolic Content Quantification Assay Kit (BioQuoChem, Spain) in a 20% (w/v) honey solution in a 96-well microplate according to the manufacturer’s instructions. Gallic (GAE) acid was used as the reference standard and results were expressed as GAE equivalents (mg/ml). Absorbance was measured at 700 nm at 37 °C.

### Detection of GOX and Def-1 by immunoblotting

Aliquots (15 μl) of 50% (w/v) honey solution with or without proteinase K were resolved by sodium dodecyl sulphate-polyacrylamide gel electrophoresis (SDS-PAGE) and 16.5% Tricine-SDS-PAGE using a Mini-Protean II electrophoresis cell (Bio-Rad). The proteins were transferred onto a 0.22-μm nitrocellulose Advantec membrane (Sigma-Aldrich) in 48 mM Tris, 39 mM glycine and 20% methanol using the semi-dry blotting procedure. The membrane was blocked for 1 h in a Tris-buffered saline-Tween (TBST) buffer (50 mM Tris–HCl, pH 7.5, 200 mM NaCl, and 0.05% Tween 20) containing 5% non-fat dried milk and incubated overnight with a rabbit polyclonal antibody against honeybee GOX or Def-1 (1:2000 in TBST). After washing with TBST, the membranes were incubated for 2 h in blocking buffer containing goat anti-rabbit horseradish peroxidase-linked antibodies (1:2500 in TBST; Promega). Immunoreactive bands were detected in solution containing dissolved SigmaFast 3,3-diaminobenzidine tablets (Sigma-Aldrich) or detected using enhanced chemiluminescence Immobilon Western kit (Millipore, MA, USA).

### Statistical analysis

A general linear mixed model was used to analyse the differences in MIC values following treatment with catalase and proteinase K as well as untreated samples. As every sample was tested for each treatment and both *P. aeruginosa* and *S. aureus*, the identity of samples was controlled by including a random part of the model with the sample ID. This approach allowed for the exclusion of inter-sample variability. An R programming and statistical environment was used for statistical analyses (www.R-project.org). The general linear mixed model was analysed with the lme4 package^41^. Correlations among all parameters were tested with the Hmisc package (R package version 4.1-1) using non-parametric Spearmans rank correlations. Correlations with correlation coefficient r > 0.7 or <−0.7 were considered important.

## Electronic supplementary material

Supplementary Figures
