# Supplementary material for: Phytochemicals-mediated production of hydrogen peroxide is crucial for high antibacterial activity of honeydew honey
Source: Sci Rep. 2018 Jun 13;8:9061. doi: 10.1038/s41598-018-27449-3 (PMC5998132; doi:10.1038/s41598-018-27449-3)
Supplement: Supplementary file 1 — Supplementary Figures [file 41598_2018_27449_MOESM1_ESM.doc]

**Phytochemicals-mediated production of hydrogen peroxide is crucial for high antibacterial activity of honeydew honey**

Journal: Scientific Reports

Marcela Bucekova^1^, Monika Buriova^1^, Ladislav Pekarik^2^, Viktor Majtan^3^, and Juraj Majtan^1^

^1^ Laboratory of Apidology and Apitherapy, Institute of Molecular Biology, Slovak Academy of Sciences, Dubravska cesta 21, 845 51 Bratislava, Slovakia

^2^ Plant Science and Biodiversity Center, Slovak Academy of Sciences, Dubravska cesta 9, 845 23, Bratislava, Slovakia

^3^ Department of Microbiology, Faculty of Medicine, Slovak Medical University, Limbova 12, 833 03, Bratislava, Slovakia

Correspondence should be addressed to Dr. Juraj Majtan, Institute of Molecular Biology, Slovak Academy of Sciences, Dubravska cesta 21, 845 51 Bratislava, Slovakia. Phone: +421-2-59307438, Fax: +421-2-59302646, E-mail: [juraj.majtan@savba.sk](mailto:juraj.majtan@savba.sk)

**Supplementary Fig. 1**

Antibacterial activity of honeydew honey samples (n = 23) and medical-grade manuka and kanuka honey against *Staphylococcus aureus* and *Pseudomonas aeruginosa* isolates. Activity was determined with a minimum bactericidal concentration (MBC) assay. The MBC was defined as the lowest concentration of honey solution (%) with no survival viable bacteria. K, kanuka honey; M, manuka honey.


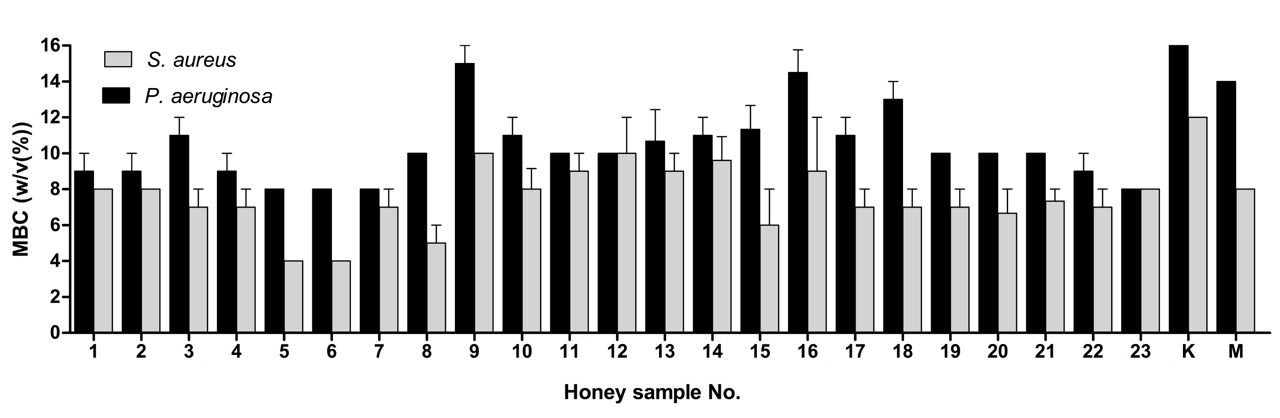


**Supplementary Fig. 2 showing full-length western blots related to Figure 4**

**
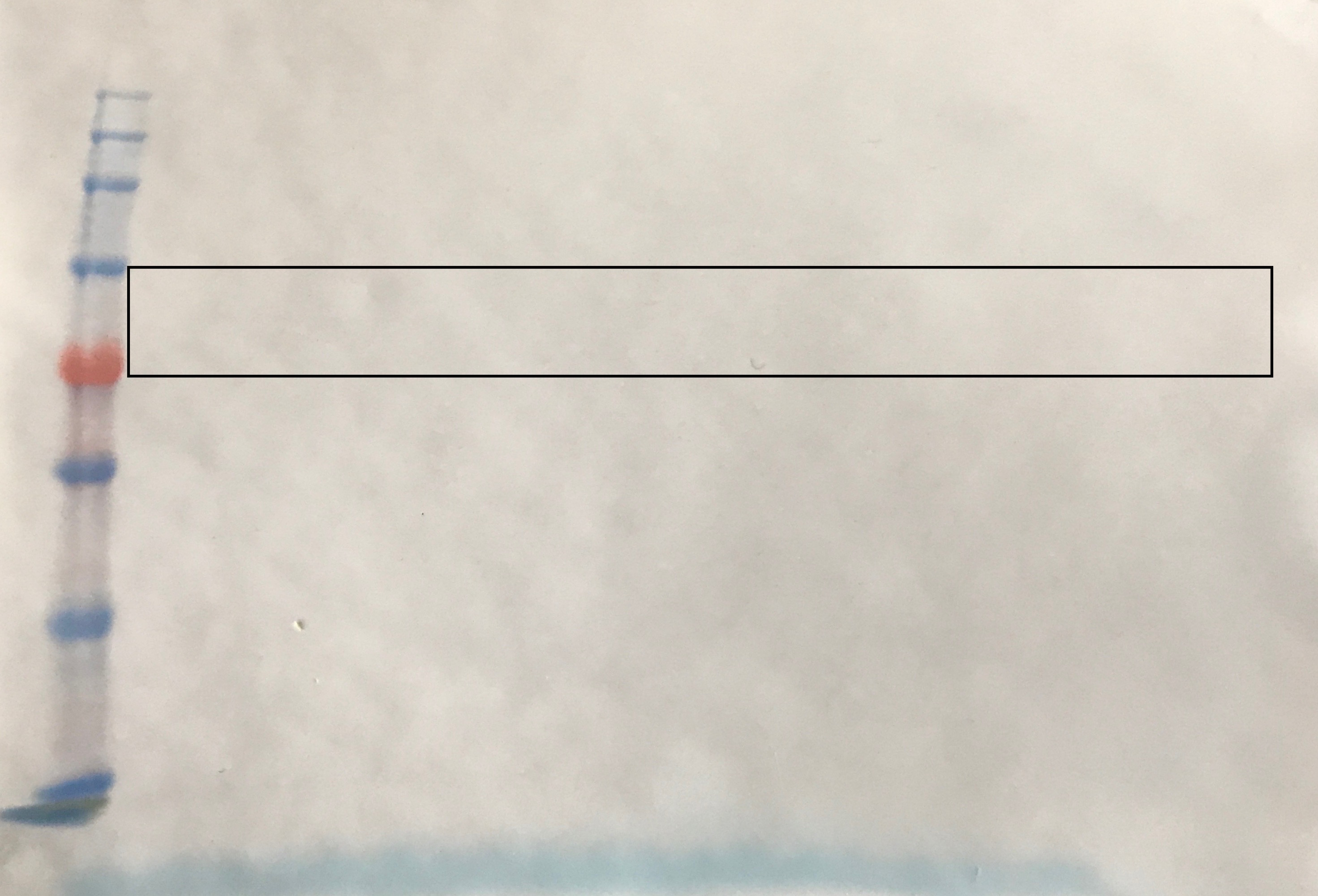

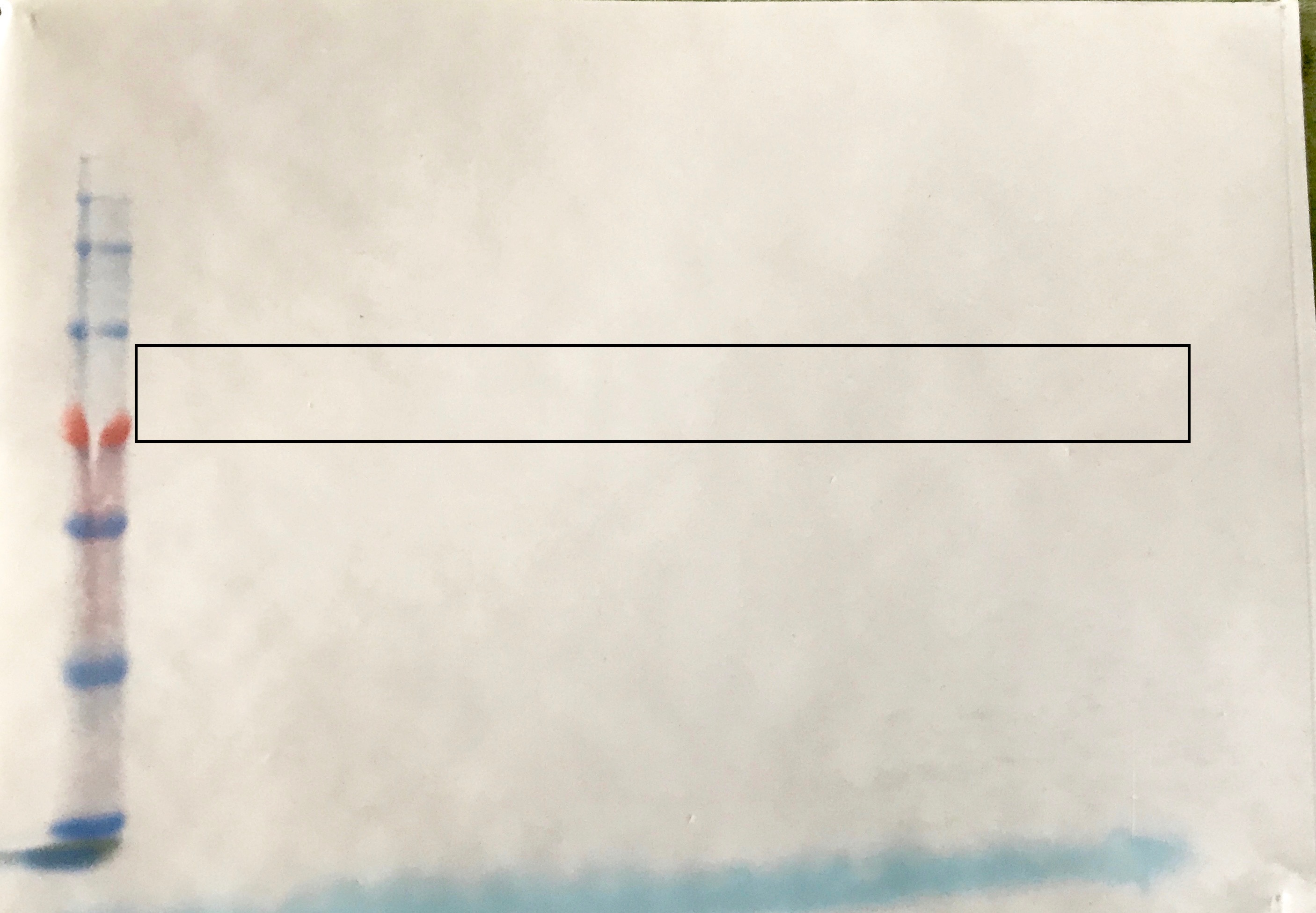
**

**Honey samples treated with proteinase-K (GOX detection by Western blot)**

**
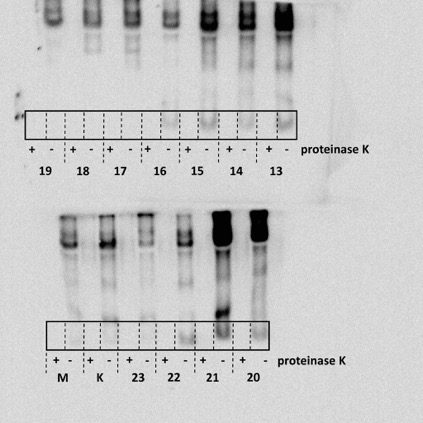

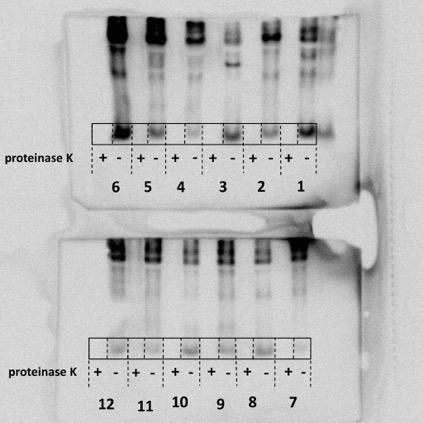
**

**Honey samples treated/untreated with proteinase K (Def-1 detection by Western blot)**

**
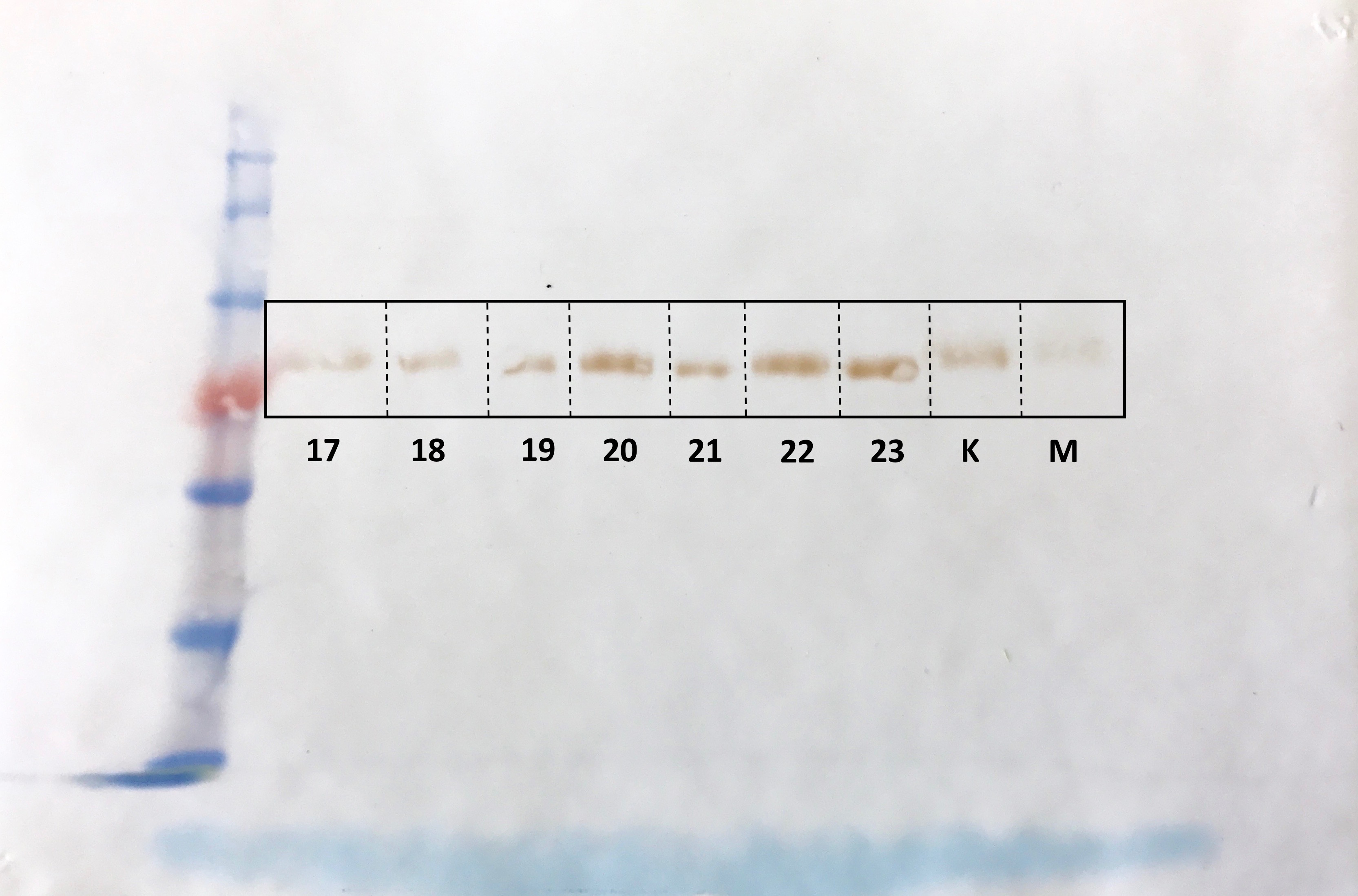

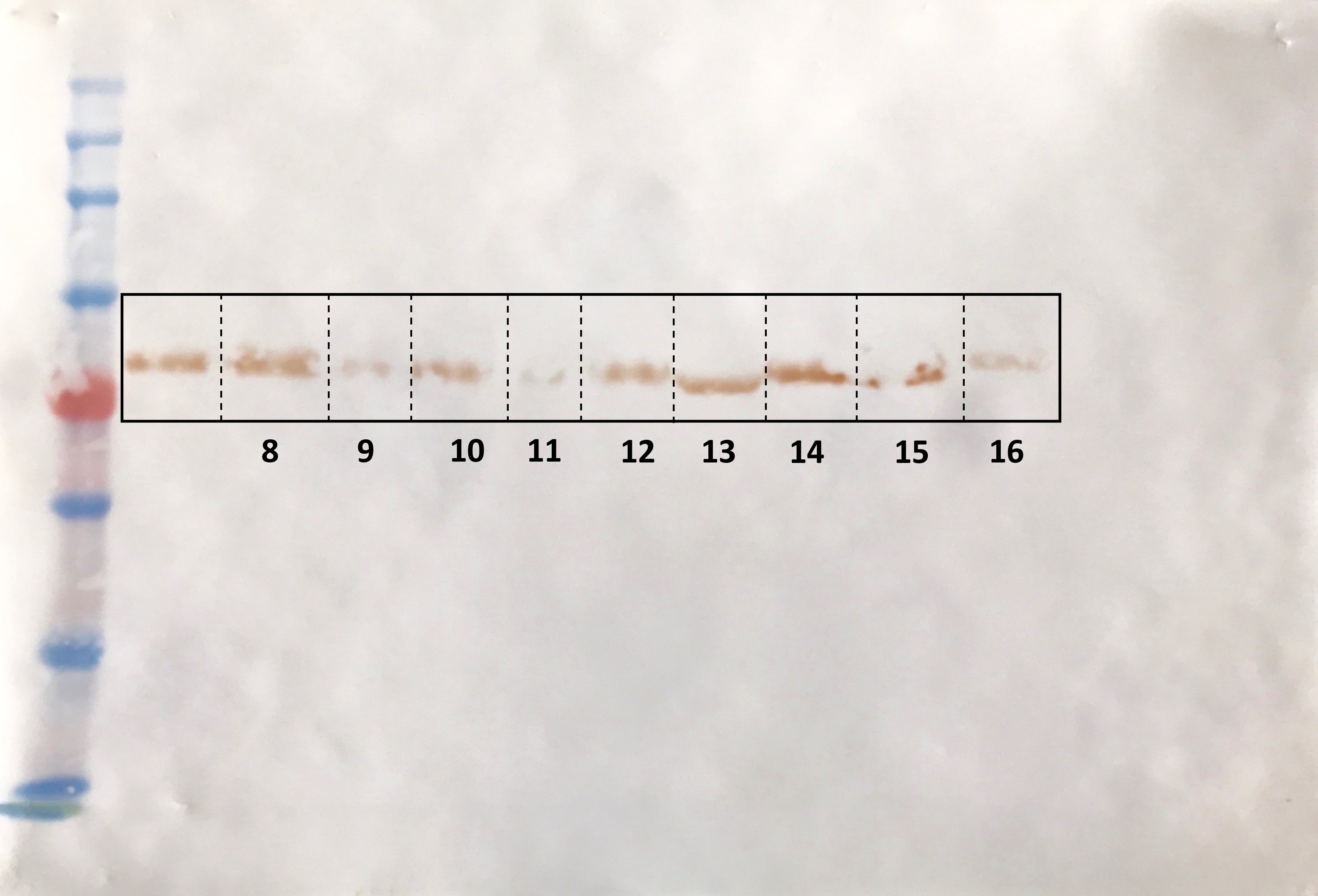

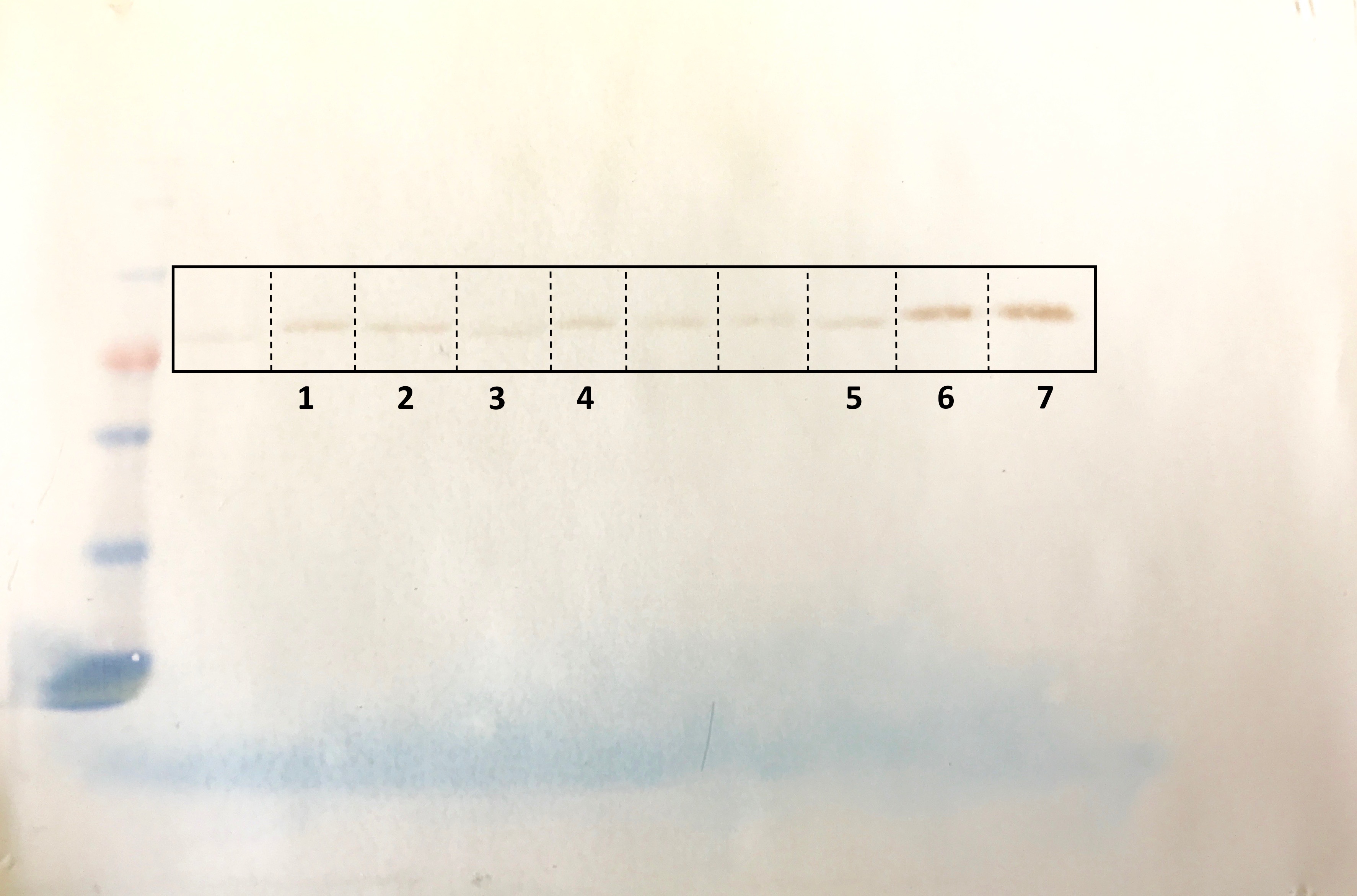
**

**Honey samples untreated (GOX detection by Western Blot)**
